# Association of Obesity and Hypertension: A Cohort Study in China

**DOI:** 10.1155/2021/1607475

**Published:** 2021-12-10

**Authors:** Xia Li, Huiqin Niu, XiaoGang Bai, YuWei Wang, Weihua Wang

**Affiliations:** ^1^Department of Endocrine and Metabolism, Yan'an University Affiliated Hospital, Yan'an, Shaanxi, China; ^2^Department of Endocrinology, Cadre Ward, Gansu Provincial People's Hospital, Lanzhou, Gansu, China; ^3^Shaanxi Provincial Center for Disease Control and Prevention, Xi'an, Shaanxi, China

## Abstract

The prevalence of general and central obesity has increased rapidly in China for decades, while little is known on obesity-normal weight-central obesity (NWCO) in China. In this study, we aim to depict the trend of the three kinds of obesity and to explore their associations with hypertension in a cohort study in China. We used data from eight waves of the China Health and Nutrition Survey (CHNS) in 1993, 1997, 2000, 2004, 2006, 2009, 2011, and 2015 for analysis. The Cochran–Armitage test was used for trend of the three kinds of obesity or hypertension. Mixed logistic regression was used to explore their relationship. In this study, we found the prevalence of general obesity increased from 20.81% in 1993 to 50.57% in 2015 in China, which was from 19.23% to 56.15% for central obesity and from 27.20% to 49.07% for NWCO, respectively. Males had the highest increase among all the subgroups. The RR for hypertension and general obesity was 3.71 (95%CI: 3.26–4.22), 3.62 (95%CI 3.19–4.12) for central obesity, and 1.60 (95%CI 1.23–2.06) for NWCO after adjusted for age, sex, education, smoking, alcohol drinking, marriage status, urbanicity and income. Both prevalence of obesity and hypertension have increased significantly in China for the two decades. The general obesity was most likely to develop hypertension compared to central or NOCWO in this study.

## 1. Introduction

Obesity has become a worldwide public health threat which is the risk factor of many chronic diseases, such as cardiovascular disease, cancer, diabetes, and chronic kidney diseases [1, 2]. There are several factors associated with obesity, such as heredity and disease complications. However, a group of evidence has showed that obesity could be attributed to life styles, for example, dietary pattern, physical activity, smoking, and alcohol drinking [3–5]. With rapid development in economics for decades, the Chinese population has shifted its traditional lifestyles to a more western way, which may be accompanied with more calorie intake in dietary and sedentary lifestyle in workplace and from home [6, 7]. All these factors resulted in an increasing prevalence of obesity. Therefore, the prevalence of obesity in China has increased dramatically from 15.8 to 30.3% between 1993 and 2011 [8].

Body mass index (BMI), which is defined as weight divided by the square of the height (kg/m^2^), is the most common indicator to identify general obesity in adults. Waist circumference (WC), which could consider body fat distribution, is used to evaluate abdominal obesity. It has more significant relationship with disease burden [9, 10]. Obesity-normal weight-central obesity (NWCO) combined general obesity and abdominal obesity has drawn more attention [11, 12]. Evidence has showed NWCO was positively associated with systemic inflammation, cardiometabolic dysregulation, and mortality [12, 13]. Therefore, we use BMI, WC, and NWCO to define general, abdominal obesity, and normal weight with central obesity in this study, respectively.

The optimal predictive obesity indicators for noncommunicable chronic diseases varied among researches. However, these three indicators of obesity negatively associated with various diseases were certain. Growing evidence has shown the positive association of obesity with hypertension. For example, central obesity is a more risk predictor than general obesity for hypertension [14–16]. However, few studies showed the association of NWCO and hypertension, as well as their magnitude.

Therefore, we describe the secular trend of prevalence of three types of obesity, as depicted by BMI, WC, and NWCO and the prevalence of hypertension. We also explored their relationships, especially the magnitude in a large cohort study of China, to provide perspectives for health interventions on obesity and hypertension.

## 2. Methods

### 2.1. Study Design and Population

The CHNS was a cohort study on nutrition status and health change among Chinese adults. A multistage, cluster, random sampling method was adopted for the study in China (Liaoning, Shandong, Henan, Jiangsu, Hubei, Hunan, Guizhou, and Guangxi). The study was conducted ten waves from 1989 to 2015. All families and individuals recruited for the first time were invited to participate the follow-ups. Details of the study were published elsewhere [17, 18]. Height, weight, waist circumference, hip circumference, and blood pressure were measured in the survey from 1993 to 2015. Hence, we used data of CHNS from 1993 to 2015 to explore the associations.

**Figure 1:**
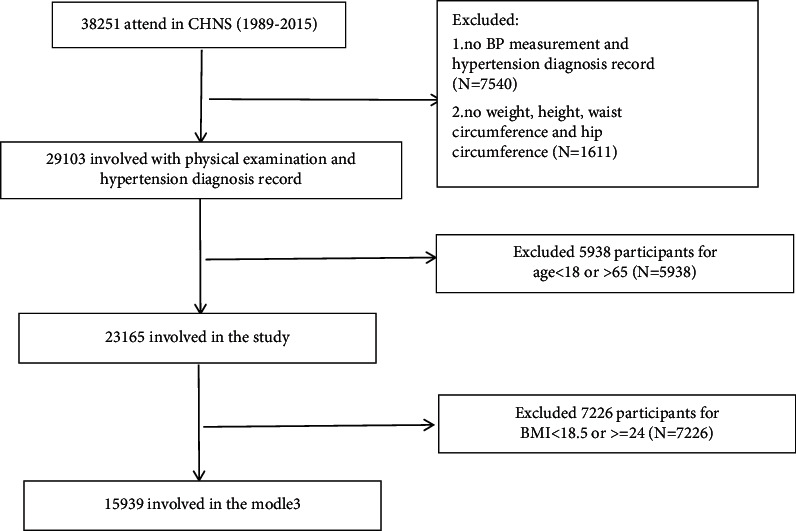
Flow chart of participants enrolled in this study measurements.

We included participates aged 18–65 in this study. Participants missed key information on BP measurement or hypertension diagnosis record (*N* = 7540), as well as measurement of weight, height, waist circumference, and hip circumference (*N* = 1611), were also excluded. Other missing data were imputed with a multiple imputation method. A total of 23165 participants were involved in the final analysis. The flow chart is shown in Figure 1.

The National Institute of Nutrition and Food Safety (Beijing, China) and the institutional review committees of the University of North Carolina (Chapel Hill, NC, USA) approved the study. Informed consent was obtained from each participant before the survey.

Weight, height, WC, and BP were measured following standardized protocols from the World Health Organization (WHO) [19]. Weight was measured with the participants wearing light clothing on a calibrated beam scale, and height was measured without shoes using a portable stadiometer. BMI was calculated as weight (kilogram) divided by squared height (meter), rounded to the nearest tenth. WC was measured with an inelastic tape to the nearest 0.1 cm at a midpoint between the bottom of the rib cage and the top of the iliac crest at the end of exhalation. HC was measured at the level of maximal gluteal protrusion using a SECA tape to the nearest 0.1 cm. BP was measured by trained examiners using a mercury sphygmomanometer at three different consecutive times with 3–5 min intervals on one visit. The three readings were averaged as the BP values in our data analysis. All physical examinations were performed at the same location and followed the same protocol at each study visit.

### 2.2. Definition

General obesity was defined as a BMI ≥28 kg/m^2^, and central obesity was defined as WC ≥ 85 cm for men and ≥80 cm for women, which is recommended by the Working Group on Obesity in China [20]. Therefore, we classified obesity into three types: general obesity (BMI ≥28 kg/m^2^), central obesity (WC ≥ 85/80 cm), and NWCO (24 > BMI ≥ 18 kg/m^2^ and waist height ratio ≥ 0.5 or waist to hip ratio ≥ 0.9/0.85 or waist>85/80 cm). Participants with BMI <28 kg/m^2^ and WC < 90/80 cm were regarded as normal in this study. Hypertension was defined as a self-reported doctor diagnosis of hypertension, a measured mean systolic blood pressure (SBP) ≥ 140 mmHg or diastolic blood pressure (DBP) ≥90 mmHg [21].

### 2.3. Covariate

Demographical variables in the study included age (18–35, 36–50, and 51–65 years), sex, marital status, education, ethnicity, residency, and household income. We categorized marital status into currently married and unmarried (single/divorced/widowed). Education was classified into low, middle, and high. Sex (male/female) and residency (urban/rural areas) were also included in the analysis.

Participants who smoked cigarettes or drank alcohol during the previous year were recognized as smokers (including former smokers and current smokers) or drinkers (including former drinkers and current drinkers), separately. In this study, the outcome variables were the prevalence of three types of obesity (general obesity, central obesity, and NWCO) and hypertension.

### 2.4. Statistical Analysis

We used mean (standard deviation) for continuous data normally distributed and median ± QD for continuous data abnormally distributed and frequency (percentage) for categorical data. The *T* test or one-way ANOVA and *χ*^2^ test were used to compare the difference between groups, respectively.

To explore the relationship between the 3 types of obesity and hypertension, we established a set of models using mixed logistic regression. Model 1 was adjusted for age and sex; model 2 was further adjusted for education, smoking, and alcohol drink; and model 3 was further adjusted for income. All analyses were completed with *R* 3.4.3. *P* < 0.05 was considered statistically significant in this study.

## 3. Results

From 1993 to 2015, 23165 participants with 66744 interviews were enrolled in this study. More female participants than the male were recruited at each survey among the 8 waves. The median age of all the participants increased from 38.00 (28.00, 48.00) to 50.00 (40.00, 58.00). The proportion of married participants increased from 80.36% to 89.97% from 1993 to 2015, as participants from urban areas and participants with low education level. Proportion of current smokers decreased from 32.11% to 24.24% during the 8 waves of survey, so does the proportion of alcohol drinking. The body weight, waist circumference, and hip circumference of participants from 1993 to 2015 increased from 55.00 kg to 62.80 kg, 74.00 cm to 84.20 cm, and 89.00 cm to 95.30 cm, respectively, as shown in Table 1.

The prevalence of general obesity increased from 20.81% in 1993 to 50.57% in 2015, in which male were from 17.24% to 53.32% and female were from 24.01% to 48.22%, respectively. The prevalence of central obesity increased from 19.23% to 56.15% during the 8 waves of survey, male from 14.80% to 56.70% and female from 23.20% to 55.68%. The prevalence of NWCO was 27.20% in 1993 and 49.07% in 2015, in which 20.39% was for male and 33.93% was for female in 1993 and 45.97% was for male and 51.49% was for female in 2015. Meanwhile, the general prevalence of hypertension increased from 13.11% in 1993 to 32.14% in 2015, male from 14.86% to 37.09% and female from 11.54% to 27.92%. Participants aged 51–65 years showed the highest increase from 31.40% to 45.55%, as shown in Table 2.

The associations between the 3 types of obesity and hypertension are presented in Table 3. We adjusted the potential confounders step by step to test the robust of the associations. The RRs was consistent for general obesity and hypertension from model 1 to model 3 (model 1: RR = 3.46, 95%CI 3.24–3.70; model 2: RR = 3.46, 95%CI 3.24–3.70; and model 3: RR = 3.71, 95%CI 3.26–4.22). The RR for central obesity and hypertension was 2.89 (95%CI 2.71–3.08) in model 1 and 3.62 (95%CI 3.19–4.12) in model 3. The association between NWCO and hypertension was 1.60 (95%CI 1.23–2.06) after we adjusted for age, sex, education, smoking, alcohol drink, marriage status, urbanicity, and income.

## 4. Discussion

Our study demonstrated that the prevalence of general obesity, central obesity, and NWCO increased significantly in China from 1993 to 2015. Attention should be drawn that all of the highest increases of the three types of obesity were among the male in this study. The prevalence of hypertension increased nearly 20% among Chinese adults from 1993 to 2015. Participants with general and central obesity had risk of incident hypertension, whereas those who were NWCO also had risk to develop hypertension.

The study showed that the obesity increased significantly in China during the two decades. The prevalence of general obesity increased from 20.81% to 50.57% in China, which is in line with previous studies [22, 23]. Male had the highest increase compared to their counterparts in our study. This finding is similar to the study in Korean men, whose general obesity increased from 22.1% to 27.5% in 10 years [24]. The unhealthy and irregular diets with high calories and socializing with alcohol binge plus with sedentary lifestyle may attribute to the high increase of general obesity, especially among male residents [25, 26]. The high increase of obesity among the old-age group also needed to be noticed, which could possibly place a heavy disease burden related to obesity in the future. We observed the same trend in central obesity, and central obesity had the highest increase among the three types of obesity, which is similar to other East Asian countries [24, 27]. What is worthy of attention is that the prevalence of NWCO also increased from 20.39% in 1993 to 45.97% in 2015, which is more than doubled. Seldom studies have the report; however, NOWCO is often ignored, but is a risk to heath. Some studies found that NWCO was associated with hypertension, diabetes, insulin resistance, low HDL, and high TG [28, 29]. Public health policy should be made to prevent and control NOWCO besides general or central obesity.

Meanwhile, we found in this study that the prevalence of hypertension increased more than doubled from 13.11% in 1993 to 32.14% in 2015. Other studies in China also revealed the high increase of prevalence of hypertension [30, 31]. However, it is estimated that the prevalence would grow 55% by year 2050 if no interventions on hypertension control are taken [32]. That is a huge challenge for China, economically and socially. It is suggested that further public policies and measures on hypertension should be implemented.

We also investigated the relationship between the three types of obesity and hypertension in this study. The association between obesity and hypertension has been widely reported [33–35]. However, few studies compared their different associations of the three types of obesity in one study, particularly the association with NWCO. The results in this study have demonstrated that the risk of incident hypertension was general obesity, followed by central obesity and NWCO. Few studies on this topic were available [8]. Due to the different definition of obesity, the order of RR is different from the previous one. However, attention should be paid to NWCO besides the other two types of obesity.

There are several limitations in this study. First, the latest released data of CHNS are wave of 2015; thus, we could not show the updated results in this study. Second, some time-dependent variables could not control in the models since covariates adjusted in the models were investigated in baseline survey. Third, the trends in this study were only China-representative, no subnational level or provincial level could conclude in this study. Last, the proportion of senior citizens was large with the wave, so more young ones should recruit in the following waves of the survey.

In conclusion, we found that both prevalence of obesity and hypertension have increased significantly in the two decades. The general obesity was most likely to develop hypertension compared to central or NOCWO in this study.

## Figures and Tables

**Table 1:** Characteristics of the eight waves of survey.

All participants	1993 (*n* = 6583)	1997 (*n* = 7363)	2000 (*n* = 8061)	2004 (*n* = 8212)	2006 (*n* = 8020)	2009 (*n* = 8318)	2011 (*n* = 11059)	2015 (*n* = 9128)
*Sex (%)*								
Male	3109 (47.23)	3582 (48.65)	3845 (47.7)	4067 (49.53)	3946 (49.2)	4061 (48.82)	5297 (47.9)	4203 (46.05)
Female	3474 (52.77)	3781 (51.35)	4216 (52.3)	4145 (50.47)	4074 (50.8)	4257 (51.18)	5762 (52.1)	4925 (53.95)
Age, *M* (IQR)	38.00 (28.00, 48.00)	40.00 (29.00, 49.00)	41.00 (31.00, 50.00)	44.00 (34.00, 53.00)	45.00 (36.00, 54.00)	46.00 (37.00, 55.00)	47.00 (38.00, 56.00)	50.00 (40.00, 58.00)

*Marriage status (%)*								
Married	5262 (80.36)	5945 (81.29)	6469 (83.22)	7048 (86.16)	7029 (87.71)	7264 (87.37)	9710 (87.84)	8212 (89.97)
Single/divorced/widowed	1272 (19.43)	1367 (18.69)	1301 (16.74)	1127 (13.78)	971 (12.12)	1017 (12.23)	1289 (11.66)	898 (9.84)
Unknown	14 (0.21)	1 (0.01)	3 (0.04)	5 (0.06)	14 (0.17)	33 (0.4)	55 (0.5)	17 (0.19)

*Urbanicity (%)*								
Urban	2076 (31.54)	2573 (34.94)	2648 (32.85)	2656 (32.34)	2501 (31.18)	2570 (30.9)	4313 (39)	3433 (37.61)
Rural	4507 (68.46)	4790 (65.06)	5413 (67.15)	5556 (67.66)	5519 (68.82)	5748 (69.1)	6746 (61)	5695 (62.39)

*Education (%)*								
Low	3573 (54.28)	4138 (56.2)	4755 (58.99)	4918 (59.89)	4363 (54.4)	4816 (57.9)	5668 (51.25)	5640 (61.79)
Middle	1044 (15.86)	1316 (17.87)	1567 (19.44)	1767 (21.52)	1822 (22.72)	1706 (20.51)	2438 (22.05)	2192 (24.01)
High	117 (1.78)	192 (2.61)	347 (4.3)	324 (3.95)	437 (5.45)	445 (5.35)	1429 (12.92)	1284 (14.07)
Unknown	1849 (28.09)	1717 (23.32)	1392 (17.27)	1203 (14.65)	1398 (17.43)	1351 (16.24)	1524 (13.78)	12 (0.13)

*Smoking (%)*								
Never	0	0	0	9(0.11)	9(0.11)	1(0.01)	1(0.01)	1(0.01)
Former	17(0.26)	19(0.26)	40(0.50)	227(2.76)	257(3.20)	225(2.70)	388(3.51)	194(2.13)
Current	2114(32.11)	2348(31.89)	2453(30.43)	2526(30.76)	2361(29.44)	2464(29.62)	3083(27.88)	2213(24.24)
Alcohol (%)	2390(36.31)	2723(36.98)	2834(35.16)	2923(35.59)	2800(34.91)	2993(35.98)	4093(37.01)	2645(28.98)
Weight, *M* (IQR)	55.00 (50.00, 61.70)	56.50 (50.90, 63.50)	58.30 (52.00, 65.10)	59.20 (52.50, 66.50)	60.00 (53.00, 67.00)	60.40 (53.80, 68.38)	61.90 (55.00, 70.00)	62.80 (55.50, 71.20)
Height, *M* (IQR)	160.00 (154.10, 166.00)	160.10 (154.80, 166.55)	160.80 (155.10, 167.10)	161.00 (155.50, 167.50)	161.30 (156.00, 168.00)	162.00 (156.00, 168.00)	162.00 (156.20, 168.30)	161.90 (156.00, 168.00)
WC, *M* (IQR)	74.00 (69.00, 81.00)	76.00 (71.00, 83.00)	78.00 (72.00, 85.00)	80.00 (73.27, 87.00)	80.00 (74.00, 87.00)	82.00 (75.00, 89.00)	83.00 (76.00, 90.30)	84.20 (77.00, 92.00)
HC, *M* (IQR)	89.00 (85.00, 94.00)	91.00 (86.00, 96.00)	93.00 (88.00, 98.00)	93.00 (88.00, 98.00)	93.00 (88.50, 98.00)	94.00 (89.00, 99.20)	95.00 (90.00, 100.00)	95.30 (90.20, 100.30)
Incoming, Mean ± SD	2263.35 ± 2175.66	5212.44 ± 3886.57	6918.96 ± 8610.28	3674.05 ± 8151.15	4742.14 ± 12251.47	7974.06 ± 19723.31	14079.23 ± 27782.74	28255.24 ± 225484.21

WC stands for waist circumference, HC stands for hip circumference, *M* stands for median.

**Table 2:** The prevalence of three types of obesity and hypertension.

Outcomes (%)	1993 (*n* = 6583)	1997 (*n* = 7363)	2000 (*n* = 8061)	2004 (*n* = 8212)	2006 (*n* = 8020)	2009 (*n* = 8318)	2011 (*n* = 11059)	2015 (*n* = 9128)
I. General obesity	1370 (20.81)	1925 (26.14)	2592 (32.15)	2889 (35.18)	2965 (36.97)	3333 (40.07)	5001 (45.22)	4616 (50.57)
Male	536 (17.24)	828 (23.12)	1170 (30.43)	1374 (33.78)	1445 (36.62)	1677 (41.30)	2485 (46.91)	2241 (53.32)
Female	834 (24.01)	1097 (29.01)	1422 (33.73)	1515 (36.55)	1520 (37.31)	1656 (38.90)	2516 (43.67)	2375 (48.22)
Age18–35 years	397 (24.82)	562 (31.13)	602 (37.46)	520 (38.91)	458 (39.96)	471 (43.29)	709 (47.39)	559 (52.28)
Age 36–50 years	585 (28.14)	814 (33.29)	1211 (38.91)	1309 (41.09)	1334 (42.03)	1435 (44.65)	2019 (49.93)	1708 (54.31)
Age51–65 years	388 (20.81)	549 (26.14)	779 (32.15)	1060 (35.18)	1173 (36.97)	1427 (40.07)	2273 (45.22)	2349 (50.57)

II. Central obesity	1266 (19.23)	1815 (24.65)	2535 (31.45)	3063 (37.30)	3153 (39.31)	3793 (45.60)	5657 (51.15)	5125 (56.15)
Male	460 (14.80)	783 (21.86)	1149 (29.88)	1423 (34.99)	1476 (37.40)	1779 (43.81)	2703 (51.03)	2383 (56.70)
Female	806 (23.20)	1032 (27.29)	1386 (32.87)	1640 (39.57)	1677 (41.16)	2014 (47.31)	2954 (51.27)	2742 (55.68)
Age 18–35 years	310 (10.89)	468 (15.10)	520 (18.40)	535 (23.59)	473 (25.01)	545 (30.16)	790 (35.16)	604 (39.32)
Age 36–50 years	511 (21.68)	766 (29.29)	1157 (35.79)	1304 (38.76)	1342 (40.20)	1481 (44.68)	2228 (52.30)	1762 (53.93)
Age 51–65 years	445 (32.27)	581 (35.23)	858 (42.86)	1224 (47.44)	1338 (47.94)	1767 (55.29)	2639 (57.97)	2759 (63.79)

III. NWCO	1266 (27.20)	1512 (30.73)	1714 (34.37)	1926 (39.45)	1887 (40.59)	1992 (44.16)	2637 (47.19)	2027 (49.07)
Male	472 (20.39)	600 (24.09)	713 (29.03)	847 (34.13)	803 (34.70)	841 (38.76)	1140 (43.31)	833 (45.97)
Female	794 (33.93)	912 (37.55)	1001 (39.55)	1079 (44.96)	1084 (46.42)	1151 (49.17)	1497 (50.64)	1194 (51.49)
Age 18–35 years	405 (18.65)	486 (21.31)	459 (23.21)	451 (28.95)	351 (28.10)	334 (29.32)	479 (36.29)	249 (30.44)
Age 36–50 years	488 (29.72)	596 (35.29)	723 (37.77)	770 (39.39)	746 (39.26)	721 (41.20)	949 (44.68)	662 (45.66)
Age 51–65 years	373 (44.35)	430 (45.26)	532 (48.58)	705 (51.50)	790 (52.67)	937 (57.77)	1209 (56.39)	1116 (59.90)

Hypertension	863 (13.11)	1204 (16.35)	1418 (17.59)	1580 (19.24)	1540 (19.20)	2042 (24.55)	2626 (23.75)	2934 (32.14)
Male	462 (14.86)	669 (18.68)	763 (19.84)	905 (22.25)	869 (22.02)	1112 (27.38)	1417 (26.75)	1559 (37.09)
Female	401 (11.54)	535 (14.15)	655 (15.54)	675 (16.28)	671 (16.47)	930 (21.85)	1209 (20.98)	1375 (27.92)
Age 18–35 years	140 (4.92)	204 (6.58)	163 (5.77)	141 (6.22)	115 (6.08)	124 (6.86)	119 (5.30)	147 (9.57)
Age 36–50 years	290 (12.30)	442 (16.90)	533 (16.49)	563 (16.74)	543 (16.27)	692 (20.87)	786 (18.45)	817 (25.01)
Age 51–65 years	433 (31.40)	558 (33.84)	722 (36.06)	876 (33.95)	882 (31.60)	1226 (38.36)	1721 (37.81)	1970 (45.55)

WC stands for waist circumference; NWCO stands for normal weight with central obesity.

**Table 3:** Association between the 3 types of obesity and hypertension.

RR (95%CI)	General obesity	Central obesity	NWCO
Model 1	3.46 (3.24–3.70)	2.89 (2.71–3.08)	1.42 (1.30–1.56)
Model 2	3.31 (2.97–3.70)	3.12 (2.81–3.46)	1.48 (1.28–1.71)
Model 3	3.71 (3.26–4.22)	3.62 (3.19–4.12)	1.60 (1.23–2.06)

Model 1: adjusted for age and sex; model 2: further adjusted for education (tertiles), smoking (tertiles), alcohol drink, and marriage status; model 3: further adjusted for urbanicity and income (continuous).

## Data Availability

Data can be found on the Internet via searching for CHNS.
